# Odontalgia

**Published:** 1881-11

**Authors:** Edward H. Bowne


### ARTICLE IV.

### *Odontalgia.*

BY EDWARD H. BOWNE, D. D. S.

Burns, the eminent Scottish poet, demonstrated toothache the "hell of diseases"—an opinion agreed with by many persons, so far as the pain is concerned at least.

At the battle of Brandy Station, in the civil war, was a trooper who had suffered several days with a toothache. In a hand to hand fight he received a pistol ball in the right cheek, It knocked out his aching molar, and passed out of the left hand corner of his mouth, taking along a part of an upper tooth. The joy of being rid of the toothache was so great, that the trooper could not be made to go to the rear to have his wound dressed.

On the 20th of May 1879, the writer, by falling from a scaffolding, received a terrible fracture of both bones below the knee, the tibia and fibula. During the most painful part of the "knitting" process, the roots of the third left lower molar commenced to ache, and the pain was so great, that he utterly forgot the pain of the fractured bones, and the intensely sensitive heel.

In the history of Rhode Island, we are told, that American Indians, were known to have been burned alive, without a murmer of pain, but, nevertheless would shriek with agony when suffering from the toothache.

Notwithstanding the [sometimes] terrible agony of toothache, people still hesitate to endure the momentary pain of extraction, and will often times suffer for years, with diseased and aching teeth rather than submit to the operation.

The writer is aware of the prejudice against the use of anæsthetics in teeth extraction, still, he thinks in a large number of dental operations the use of anæsthetics are perfectly justifiable, and even necessary, to prevent the shock of an operation. Experience has demonstrated that Nitrous Oxide Gas, is the safest anæsthetic known to Surgery—properly administered, it can be used with almost absolute freedom from danger; and, we consider the discavery of anæsthetics (notwithstanding the remarks of Dr. Atkinson, of New York, at a meeting of the New Jersey Dental Society, at Long branch, July 22, 1880, that "anæsthesia is a lieutenant of his satanic majesty.") one of the greatest blessings of modern times; and we cannot close this article without quoting the language of that most classical scholar and surgeon Dr. Oliver Wendell Holmes, who in speaking of anæsthesia, exclaimed: "The knife in searching for disease, has been steeped in the waters of forgetfulness, and the deepest furrow in the knolled brow of agony has been smoothed forever."

In a lecture, Dr. John C. Warren, the eminent surgeon, in speaking of this great boon to humanity, remarded: "Who could have imagined, that drawing the knife over the delicate skin of the face might produce a sensation of unmixed delight! That the twisting and turning of instruments in the most sensitive bladder might be accompanied by a delightful dream!"

Rock Hill, N. J., August, 1881.

